# Implementing and evaluating a program to facilitate chronic disease prevention and screening in primary care: a mixed methods program evaluation

**DOI:** 10.1186/s13012-014-0135-7

**Published:** 2014-10-08

**Authors:** Donna Patricia Manca, Kris Aubrey-Bassler, Kami Kandola, Carolina Aguilar, Denise Campbell-Scherer, Nicolette Sopcak, Mary Ann O’Brien, Christopher Meaney, Vee Faria, Julia Baxter, Rahim Moineddin, Ginetta Salvalaggio, Lee Green, Andrew Cave, Eva Grunfeld

**Affiliations:** Department of Family Medicine, University of Alberta, 6-10 University Terrace, Edmonton, Alberta T6G 2T4 Canada; Covenant Health, Grey Nuns Community Hospital, 1100 Youville Drive W Northwest, Edmonton, Alberta T6L 5X8 Canada; Discipline of Family Medicine, Memorial University of Newfoundland, 300 Prince Phillip Drive, St. John’s, Newfoundland A1B 3V6 Canada; Department of Health and Social Services, Government of Northwest Territories, P.O. Box 1320, Yellowknife, Northwest Territories X1A 2L9 Canada; Department of Family and Community Medicine, University of Toronto, 500 University Ave, Toronto, ON M5G 1V7 Canada; Ontario Institute for Cancer Research, 661 University Avenue, Suite 510, Toronto, ON M5G 0A3 Canada

**Keywords:** Program evaluation, Chronic disease, Prevention, Screening, Clinical practice guidelines

## Abstract

**Background:**

The objectives of this paper are to describe the planned implementation and evaluation of the Building on Existing Tools to Improve Chronic Disease Prevention and Screening in Primary Care (BETTER 2) program which originated from the BETTER trial. The pragmatic trial, informed by the Chronic Care Model, demonstrated the effectiveness of an approach to Chronic Disease Prevention and Screening (CDPS) involving the use of a new role, the prevention practitioner. The desired goals of the program are improved clinical outcomes, reduction in the burden of chronic disease, and improved sustainability of the health-care system through improved CDPS in primary care.

**Methods/design:**

The BETTER 2 program aims to expand the implementation of the intervention used in the original BETTER trial into communities across Canada (Alberta, Ontario, Newfoundland and Labrador, the Northwest Territories and Nova Scotia). This proactive approach provides at-risk patients with an intervention from the prevention practitioner, a health-care professional. Using the BETTER toolkit, the prevention practitioner determines which CDPS actions the patient is eligible to receive, and through shared decision-making and motivational interviewing, develops a unique and individualized ‘prevention prescription’ with the patient. This intervention is 1) personalized; 2) addressing multiple conditions; 3) integrated through linkages to local, regional, or national resources; and 4) longitudinal by assessing patients over time. The BETTER 2 program brings together primary care providers, policy/decision makers and researchers to work towards improving CDPS in primary care. The target patient population is adults aged 40–65. The reach, effectiveness, adoption, implementation, maintain (RE-AIM) framework will inform the evaluation of the program through qualitative and quantitative methods. A composite index will be used to quantitatively assess the effectiveness of the prevention practitioner intervention. The CDPS actions comprising the composite index include the following: process measures, referral/treatment measures, and target/change outcome measures related to cardiovascular disease, diabetes, cancer and associated lifestyle factors.

**Discussion:**

The BETTER 2 program is a collaborative approach grounded in practice and built from existing work (i.e., *integration not creation*). The program evaluation is designed to provide an understanding of issues impacting the implementation of an effective approach for CDPS within primary care that may be adapted to become sustainable in the non-research setting.

## Background

### Introduction

The purpose of this paper is to describe the planned implementation and evaluation of the Building on Existing Tools to Improve Chronic Disease Prevention and Screening in Primary Care (BETTER 2) program. We will describe the unique approach the BETTER 2 program takes to address chronic disease prevention and screening (CDPS) in primary care settings including the frameworks and evaluation metrics that will be used to measure the effectiveness of the program in achieving its goals.

The paper is divided into the following three broad sections: 1) the ‘Background’ section provides an overview of CDPS in primary care and discusses the BETTER approach to CDPS and the integral role that the prevention practitioner plays in promoting CDPS. This section further reviews the effectiveness of the Building on Existing Tools to Improve Chronic Disease Prevention and Screening in Family Practice (BETTER) trial and describes the evolution of the BETTER 2 program including the specific objectives it seeks to accomplish. 2) The ‘Methods/Design’ section explains the prevention practitioner role and how the BETTER 2 program will expand and adapt to novel jurisdictions. This section further includes a description of the planned evaluation of the BETTER 2 program, which involves qualitative, quantitative, and health economic components. 3) Lastly, the ‘Discussion’ section highlights the unique approach utilized for knowledge integration and the potential outcomes of the proposed program evaluation.

### Background

The prevalence of chronic disease is steadily increasing [1,2]. These chronic diseases have a substantial impact on health-care services and many can be prevented. Primary care is the ideal setting for most CDPS activities. Regrettably, evidence-based tools and strategies for CDPS are inconsistently applied in primary care practice. Barriers to implementing evidence into practice occurs at multiple levels [3] and are, in part, due to competing demands on primary care physicians leaving little time to address CDPS [4,5].

**Figure 1:**
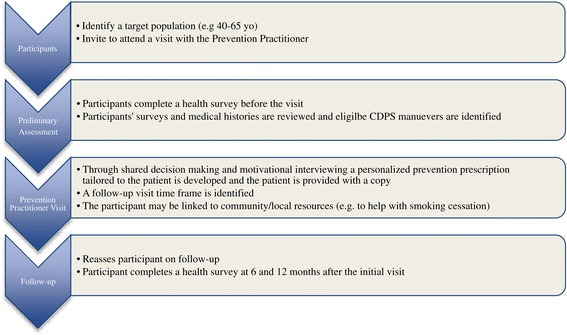
**The BETTER chronic disease prevention and screening prevention practitioner intervention.**

The BETTER trial, a pragmatic randomized controlled trial, previously demonstrated the effectiveness of an approach to CDPS that involved 1) a clinical working group (CWG) which identified and harmonized the evidence for CDPS actions in patients aged 40–65 [6], and 2) an intervention consisting of practice facilitation at the individual patient-level through the use of a prevention practitioner (Figure 1) who was internal to the practice [5,7].

The prevention practitioner was a new role introduced to the primary care practice setting [7]. In the BETTER trial, the prevention practitioners were health-care professionals (licensed practical nurse, nurse, dietician, nurse practitioner) who worked with the primary care providers to develop a comprehensive approach to evidence-based CDPS within the practice setting [5–7]. Clinicians, researchers, and prevention practitioners with support from the Center for Effective Practice in Toronto, Canada participated on the BETTER CWG which identified the evidence-based clinical guidelines and interventions that were then adapted and incorporated into the BETTER CDPS tool kit [6]. The tool kit included patient surveys, prevention visit templates, prevention prescriptions, a CDPS care map, and patient resources. The prevention practitioner utilized the BETTER CDPS tool kit to evaluate patients and, through a process of shared decision-making, provided the patient with an individualized prevention prescription and CDPS goals.

The original BETTER trial demonstrated that a prevention practitioner ‘can improve the implementation of clinically important prevention and screening for chronic diseases in a cost-effective manner’ [5] in urban multidisciplinary primary care settings in Alberta and Ontario, Canada. Further funding was obtained to disseminate, implement, and evaluate the BETTER 2 program in heterogeneous populations as well as in rural and remote settings.

### Aims of the BETTER 2 program

The overarching aims of the BETTER 2 program are to improve clinical outcomes, reduce the burden of chronic disease, and improve the sustainability of the health-care system through improved CDPS in primary care. The program targets those chronic diseases that have strong evidence for prevention and screening, specifically cancer, cardiovascular disease, diabetes, and their associated lifestyle risk factors in patients aged 40–65.

Specific objectives of the BETTER 2 program are the following:

1. To introduce the BETTER approach to new jurisdictions and deepen the impact in the two original participating provinces
2. To adapt, revise, and tailor the BETTER individual patient-level CDPS intervention including the survey, tools, actions, and resources for uptake in a variety of settings including remote, rural, aboriginal populations, and disadvantaged populations
3. To synthesize BETTER knowledge products for translation, dissemination and exchange through the development of a web resource with updated CDPS tools, actions, and training resources
4. To evaluate the adapted BETTER intervention using a mixed methods approach

## Methods/design

### Intervention (the program)

**Figure 2:**
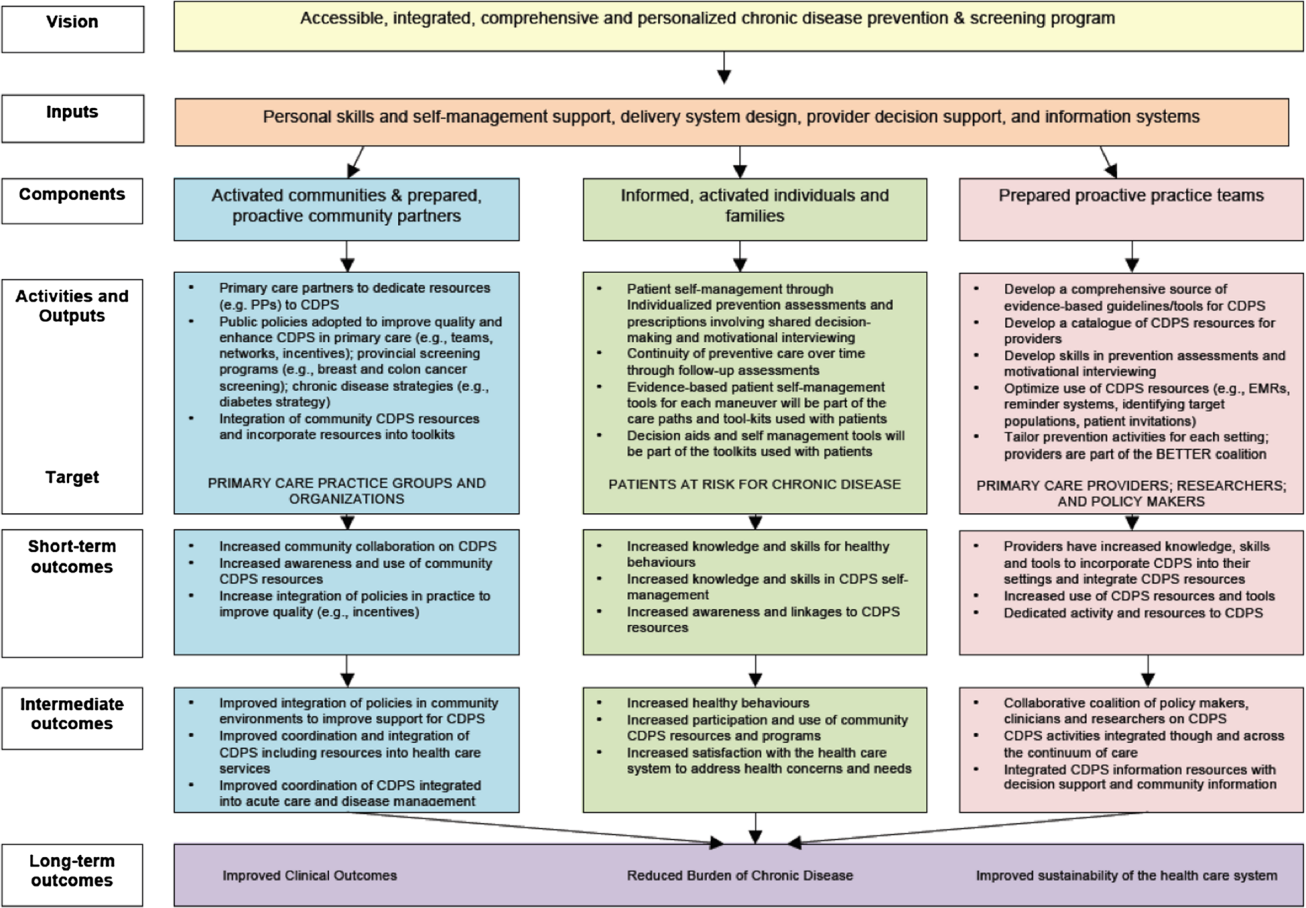
**Logic model for the BETTER 2 program.**

**Figure 3:**
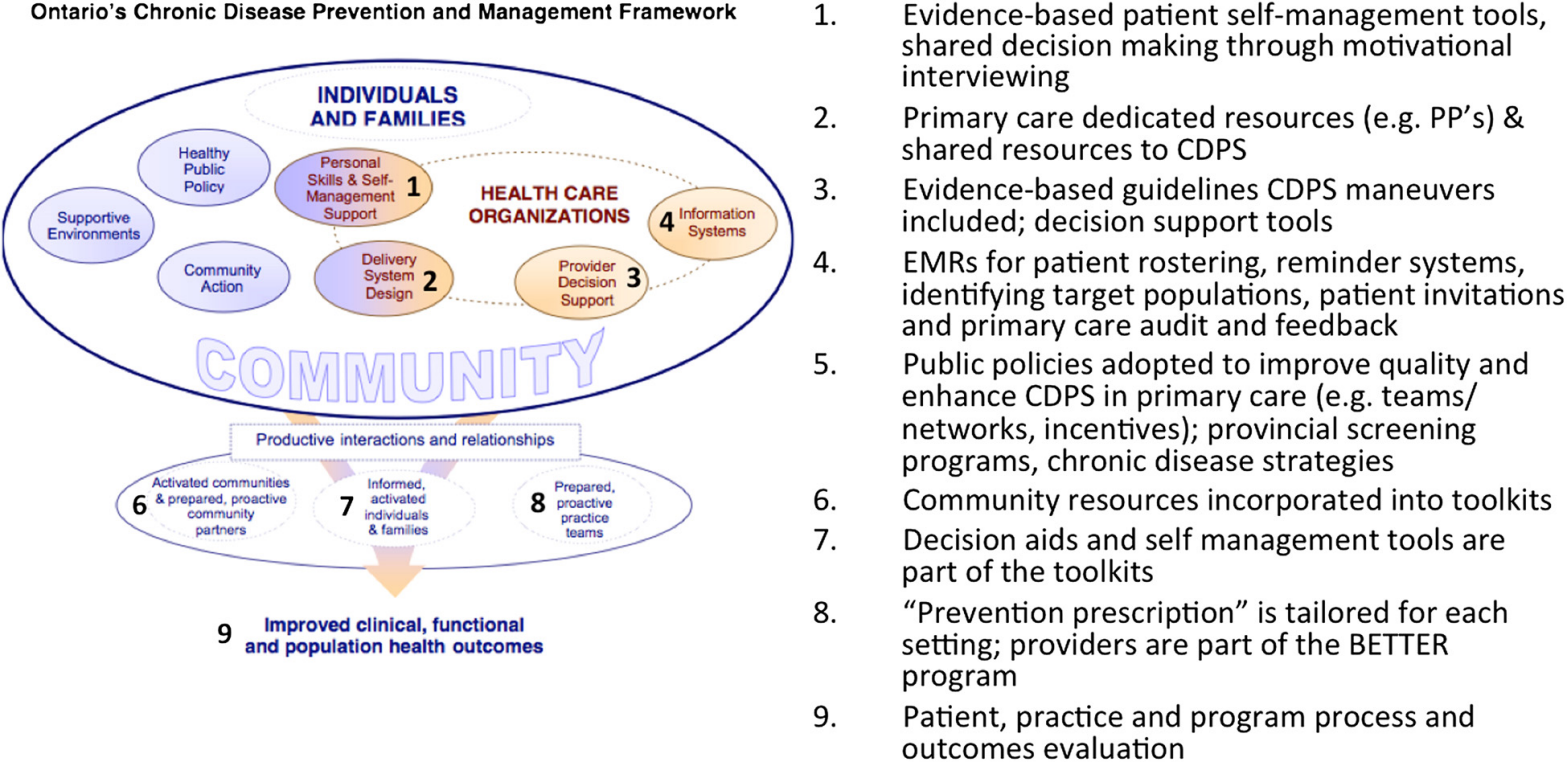
**The BETTER 2 mapped onto the chronic disease framework.**

Experts in research, practice, and policy worked together to develop the BETTER 2 program with the aim of addressing CDPS from a policy and practice perspective including the needs of the end users, such as health-care professionals, a key to integrated knowledge translation [8–11]. Since implementing a new approach and associated changes are often perceived as challenging [12], a comprehensive, multilevel approach that engages both policy makers and practitioners is needed to implement change successfully [3,13]. The Chronic Care Model is an integrated approach with multiple and multilevel strategies that has been demonstrated to be most effective in addressing chronic disease prevention and management [14–16]. This model was also adopted and described by Ontario’s Chronic Disease Prevention and Management Framework [17]. The Chronic Care Model is the foundational framework that informs the BETTER 2 program (see Figures 2 and 3). The BETTER 2 program addresses the knowledge to action gap through obtaining an understanding of the context-specific practical wisdom of the primary care providers in diverse settings and facilitates macrolevel knowledge partnerships between researchers, practitioners, and policymakers [18].

We propose to introduce an intervention that is proactive at both the population and an individual patient-level. The patient intervention consists of a dedicated prevention visit with the prevention practitioner (see Figure 1). The prevention practitioners are health professionals identified by primary care providers and trained by the BETTER 2 program to identify at-risk patients and invite them to a prevention visit. The prevention practitioners further identify local CDPS resources that are reviewed by the CWG to ensure that they are evidence based before being integrated into their BETTER 2 CDPS tool kits.

The program will collaborate with practice, policy, and research partners to expand the prevention practitioner role in Canadian provinces and territories: the Northwest Territories, Newfoundland and Labrador, and Nova Scotia, and deepen the impact in the two original participating provinces, Alberta and Ontario, through providing a framework, tools and resources for CDPS in primary care settings. To accomplish this, the BETTER 2 program, working with partners from each context, will review, revise, and adapt the original BETTER intervention, including the survey, tools, actions, and resources to each setting including urban, rural, remote settings, aboriginal populations, and disadvantaged populations. This will include identifying and cataloging CDPS resources and incentives available to the various communities engaged in the BETTER 2 program and reviewing the BETTER CDPS survey and tools to tailor them to the needs and requirements of those communities.

Presently primary care is focused on acute and chronic disease management and there is often no specific individual and too little time committed to CDPS [7]. The BETTER 2 team will support the prevention practitioners in their new role and work with them to conduct an asset mapping activity aimed to identify local, regional and national resources that the prevention practitioners can integrate into their setting and role. The BETTER 2 CWG will synthesize evidence-based CDPS knowledge products for translation, dissemination and exchange and provide a web resource [19] with updated CDPS tools and actions including training resources such as manuals, webinars and podcasts [20]. The team will also oversee and facilitate the training of the prevention practitioners to develop skills in CDPS including developing an individualized patient prescription.

### Target population

The target populations include (see Figure 2) [17] the following:

1. Activated communities and prepared, proactive community partners including primary care groups and organizations who will help to facilitate the BETTER 2 approach to CDPS through dedicating resources (such as a health-care professional’s time to take on the role of the prevention practitioner). Activated communities and partners will be the ones to support policies and organizational structures and engage in the BETTER 2 program.
2. Informed, activated individuals and families through patient participation. The sites will select and identify at-risk populations such as adult patients of the participating primary care providers who are between the ages of 40 and 65, and data will be obtained from those who provide written informed consent to participate. We chose this age group because this is the age group for which most of the CDPS actions can be applied [5].
3. Prepared proactive practice teams including primary care providers, researchers, and policy/decision makers who will work together with the BETTER 2 team to develop a comprehensive source of tools aimed to transform practice through a patient-level intervention by a health-care professional within the practice, the prevention practitioner.

### Program evaluation using the RE-AIM framework

The BETTER 2 program has developed a logic model that describes the inputs and the anticipated short-term, intermediate, and long-term outcomes (see Figure 2). The program will undergo an evaluation using the RE-AIM (reach, effectiveness, adoption, implementation, maintain) framework [21–23]. Most evaluations of health promotion impact have restricted their focus to one or two of the five dimensions of quality that are believed to be important [22]. RE-AIM provides a comprehensive framework to assess programs that work in the real-world setting [21,22].

#### Reach

The reach of the program will be captured through descriptive information. This will include information on the proportion of the patients approached, who participated, and who returned for follow-up visits including the representativeness of those participating, where possible. In the original BETTER trial, there was a 63% acceptance rate with a return rate of 81.6% at 7 months and 10% (46/444) did not attend any visits [5]. In our rural and more disadvantaged populations, we anticipate that the proportion of patients accepting and following-up on the intervention will decrease and the proportion of patients who do not show up for any visits will increase.

The reach of the program will include the following:

1. The number of patients approached to participate in the program (denominator)
2. The number of patients who agree to participate and who follow-through to actually have a visit with the prevention practitioner
3. The number of primary care providers/sites approached and educated about BETTER and the proportion that have adopted the tools or adapted the prevention practitioner role into their practices
4. Demographic information (if available) of participating patients including ethnicity, gender, age, socioeconomic status, and primary care site

#### Effectiveness

The effectiveness of the program will be evaluated using the explicitly defined summary statistics defined below as well as via the use of a more comprehensive composite index [5].

Specifically, effectiveness measures will include the following:

- The proportion of individual CDPS activities achieved at any point in time. We will identify specific CDPS action information that we can capture in the population at six-month intervals (e.g., proportion of eligible mammograms, blood pressures, alcohol history, smoking status, etc., achieved at 0, 6, and 12 months). We will track the proportion of patients completing these maneuvers longitudinally at each of these observation points.
- An evaluation of the CDPS achieved using a composite index [24] developed for CDPS and adapted from the original BETTER trial [5]. The composite index is identical in its mathematical definition to the one used in the original BETTER trial; however, the items comprising the composite are different. Informed by the learning experiences in the BETTER trial, we revised the content to get better quality data.

The composite index is comprised of process outcome measures (i.e., monitoring and screening actions), referral and treatment measures (i.e., appropriate treatment of out-of-date or off-target actions), and target/change outcomes (i.e., quantifiable change or improvement actions). The CDPS actions in each patient eligible to complete as part of the program are determined at baseline, and the achievement of these eligible actions is assessed at follow-up. Where possible, a composite index for each patient will be calculated at baseline and then at six-month intervals. If a patient is ineligible (up to date) for all CDPS maneuvers, then his/her contribution to the aggregate composite index is non-calculable as the denominator (the number of eligible items at baseline) is zero.

The composite index will allow for measures of change over time, that is, a gross indicator of effectiveness. The composite index essentially provides an average measure of effectiveness over all people enrolled in the study, and suggests the proportion of conditionally eligible maneuvers (as defined at baseline) which are achieved at follow-up. It acts to average over the heterogeneity of the patients and the maneuvers they are specifically eligible for; hence, it is referred to as a gross measure of effectiveness. For any given patient, the composite index can also act as a benchmark of how the patient is doing with respect to CDPS at any given moment in time. We will use the composite index as a comprehensive measure of CDPS to describe the proportion of eligible actions achieved at six-month intervals, and it will also allow researchers the opportunity to investigate certain comparisons of CDPS effectiveness between group factors, such as jurisdiction, age, sex, etc.

#### Adoption

Adoption will be assessed by describing the proportion, characteristics, and representativeness of Canadian settings approached and those willing to participate in the BETTER 2 program. We will describe the national/provincial/territorial characteristics of the sites, including their similarities and differences relative to other communities in a larger geographic area or region.

#### Implementation

Implementation of BETTER 2 in the diverse primary care settings will be captured by detailed description of the contextual adaptations of the program, the time required for intervention, and the costs of the intervention. In addition, the ‘Qualitative Evaluation’ section details the qualitative approach aimed to capture implementation information including process, facilitators and barriers, and contextual issues.

An economic assessment will include the following:

- A description of the implementation cost of the intervention in various settings including the amount of time the prevention practitioners spend with individual patients and the improvement in CDPS activities achieved by the various patient populations in the intense intervention groups.
- We will explore the impact of the different levels of interventions on the costs of the program and examine patterns in costs over time.

#### Maintain

We will explore the extent that the program becomes part of a routine practice and policy over time including how and if the program can be delivered over the long term. Most decisions about maintaining a program are influenced not only by the overall impact of a treatment but also by its costs [21]. The time and cost of the intervention will be captured as described above.

**Figure 4:**
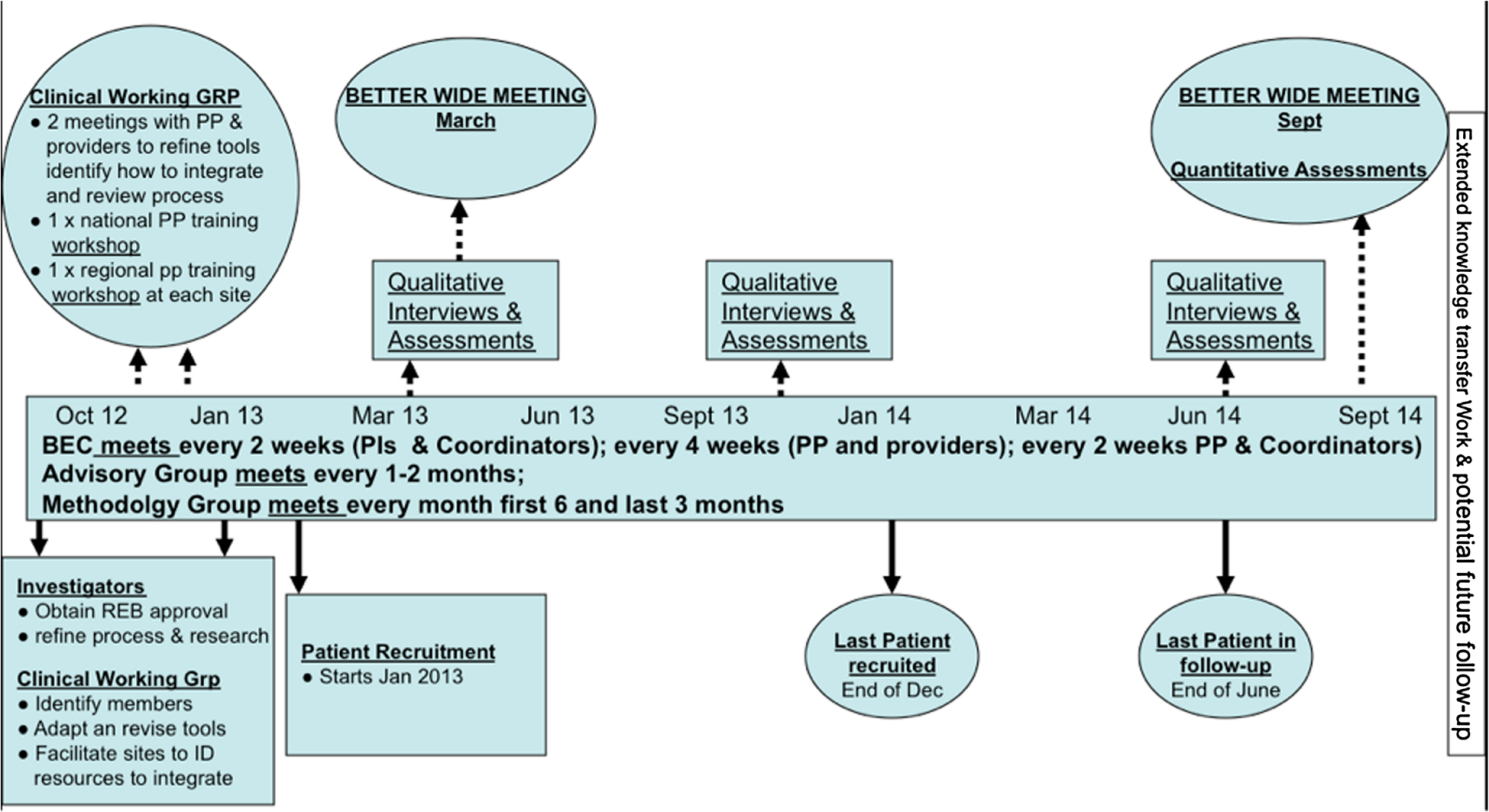
**The BETTER 2 timeline.**

Evaluation of maintenance will include the following:

- The proportion of primary care providers introduced to the BETTER 2 approach who use the BETTER tools/activities calculated at three time points (see Figure 4, the BETTER 2 timeline).
- The proportion of primary care providers introduced to the BETTER 2 approach that adapt the BETTER patient-level intervention, a prevention practitioner health-care professional, into their practices calculated at three time points (see Figure 4, the BETTER 2 timeline).

### Qualitative evaluation

A qualitative evaluation will contribute to the overall program evaluation of the BETTER 2 program and is aimed at understanding the facilitators and barriers, benefits, and disadvantages of the BETTER approach in varied settings including remote, rural, and disadvantaged populations. A qualitative evaluation will help to examine the uptake of the BETTER 2 program by involving perspectives of those who use and provide the program: health-care providers, administrators, and patients. Data collection strategies include semi-structured interviews (one-on-one interviews, focus groups, and patient feedback forms) with participants (as identified above) by a trained researcher at approximately three time periods throughout the project [25] (see Figure 4, the BETTER 2 timeline).

The goal of the BETTER 2 qualitative evaluation component is twofold. One aim is to facilitate and assist with implementation through the early identification and intervention of potential barriers to the program. The secondary goal is to determine the approach’s adaptability, sustainability, and perceived impact in primary care settings. We will capture how the approach was implemented and adapted to various settings, the impact on patients and practice including any perceived benefits or disadvantages, the feasibility of the approach, and how and if the approach will be sustained. We will explore how the tools/activities were adapted to various primary care settings including perceptions of how well these adaptations worked. We will also consider the contextual factors of the implementation of the program, specifically barriers and enablers to program implementation.

The primary qualitative research questions are

1. What is the impact of BETTER 2 on the health setting in each community?
2. How has BETTER 2 been adapted in each community?
3. What barriers and enablers are key to implementation of BETTER 2?
4. How can BETTER 2 be improved?

### Theoretical frameworks

The BETTER 2 program involves implementation of a CDPS approach into various clinical settings which can be impacted by the health system at various levels, that is, 1) national and provincial (macro), 2) regional or program (meso), and 3) site or clinic (micro) levels [26]. There are numerous models available to inform dissemination and implementation of research [3,27]; however, many miss important constructs or address only one level. Upon reviewing several frameworks, we identified three that could inform our qualitative evaluation at the macro, meso, and micro health system levels including the theoretical domains framework (TDF) [28,29], the consolidated framework for implementation research (CFIR) [3], and the awareness, desire, knowledge, ability and reinforcement (ADKAR) model [30].

The TDF is a comprehensive framework that includes all of the important constructs of implementation [28,29]. Since it is inclusive and addresses a large number of domains (14) and constructs (84), it may not be the best tool to identify and prioritize the key elements of the implementation. However, an awareness of the constructs in the TDF will help ensure that no important construct is missed during the qualitative evaluation. The CFIR is composed of five major domains that capture the characteristics of the 1) intervention, 2) outer setting, 3) inner setting, 4) individuals involved, and 5) process of implementation [3]. The CFIR framework is a pragmatic synthesis of several frameworks and models and will inform the implementation process by identifying key elements in the program implementation in a systematic way. At the micro level, the qualitative evaluation will be informed by the ADKAR model, a model that focuses on change processes on the ‘people level’ and is commonly used in business, government, and community settings [30].

### Trial status

The BETTER 2 is a program evaluation and not a research trial. The BETTER 2 program has received approval for the various research components of the program evaluation through the University of Alberta, and St. John’s Newfoundland ethics boards. The program also has a scientific research license with the Northwest Territories. The registration number of the original RCT BETTER trial was ISRCTN07170460.

## Discussion

The prevalence of chronic disease is steadily increasing [1,2], and this epidemic of chronic disease threatens the sustainability of health-care systems internationally. The BETTER trial, a pragmatic randomized controlled trial, demonstrated the effectiveness of a CDPS intervention that involved prevention practitioners in the primary care multidisciplinary team settings [5]. The intervention could be adapted to become sustainable in the non-research setting. The BETTER 2 program has been funded to bring together research, practice, and policy, through an approach to CDPS that includes the end users. This approach of engaging research, practice, and policy creates a better fit between the information and the needs of the users, a key to integrated knowledge translation [8–11]. This collaborative approach is grounded in practice and developed from existing work.

Our ultimate long-term outcomes are improved clinical outcomes and reduced burden of chronic disease through improved early detection of cancers (e.g., breast, cervical, and colon); lower incidence of cancers through modification of lifestyle (e.g., lung cancer); and reduction of chronic disease through prevention (e.g., diabetes, heart attack, stroke). Cost-effective analyses of the original BETTER trial demonstrated that a prevention practitioner ‘can improve the implementation of clinically important prevention and screening for chronic diseases in a cost-effective manner’ [5]. We expect that achieving these objectives using the BETTER 2 program will lead to cost savings and therefore improved sustainability of the health-care system. The program evaluation as outlined in this paper is designed to provide an understanding of the issues impacting the implementation of an effective approach for CDPS within primary care that may be adapted to become sustainable in the non-research setting. The RE-AIM framework informs our evaluation which includes a composite index to assess effectiveness in real-world settings [5,21,22,24].

## Abbreviations

ADKAR: Awareness, desire, knowledge, ability and reinforcement framework
BETTER trial: Building on Existing Tools to Improve Chronic Disease Prevention and Screening in Family Practice (a pragmatic randomized controlled research trial in family practice settings)
BETTER 2: Building on Existing Tools to Improve Chronic Disease Prevention and Screening in Primary Care (a program aimed to implement the BETTER interventions in expanded settings to improve CDPS in primary care)
CDPS: Chronic disease prevention and screening
CFIR: Consolidated framework for implementation research
CLASP: Coalitions linking action and science
CPAC: Canadian partnership against cancer
CWG: Clinical working group
TDF: Theoretical domains framework
